# Exploring Excited State Proton Transfer Dynamics upon Ultraviolet Excitation

**DOI:** 10.1021/acs.jpca.5c08622

**Published:** 2026-03-11

**Authors:** Nidhi Kaul, Alfy Benny, Vasilis Petropoulos, Michał Maj, Giulio Cerullo, Margherita Maiuri, Gregory D. Scholes

**Affiliations:** † Department of Chemistry, 168555 Princeton University, Princeton, New Jersey 08544, United States; ‡ Dipartimento di Fisica, 18981 Politecnico di Milano, Piazza Leonardo da Vinci 32, 20133 Milano, Italy; § Department of Chemistry—Ångström Laboratory, 8097 Uppsala University, Box 523, SE-75120 Uppsala, Sweden

## Abstract

Excited state intramolecular proton transfer (ESIPT) has been investigated in two prototypical systems—salicylaldehyde azine (SAA) and 1,5-dihydroxyanthraquinone (DHAQ)—using transient absorption spectroscopy upon ultraviolet excitation into the less studied higher excited (S_*n*_) manifold. Excitation with sub-30 fs pulses and broadband visible probing has allowed for direct measurement of the ESIPT rate. In conjunction with steady-state measurements and TD-DFT calculations, a complete delineation of the ultrafast photophysics has been carried out. In SAA, ESIPT remains ultrafast (∼30 fs), consistent with previous S_1_ excitation studies. Coherent vibrational beats maps reveal significant wavelength dependence, however. Theoretical analysis suggests that the observed modes and their intensities in coherent vibrational spectra are modulated by the nature of the electronically excited state. In DHAQ, the first direct observation of ESIPT presents a time-constant of ∼85 fs, and a slower component of 9 ps, akin to previous reports on double-proton transfer systems. Collectively, the results suggest that while the ultrafast ESIPT rate remains largely invariant vis-à-vis the excitation energy, the product yield, as well as accompanying coherent oscillations, may be substantively altered, owing to the existence of alternative decay pathways.

## Graphical abstract

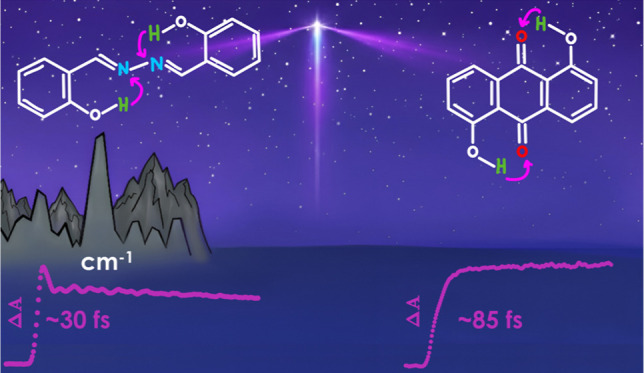

## Introduction

Among the plethora of photoinduced molecular processes in nature, a class of reactions of interest is that of excited-state intramolecular proton transfer (ESIPT). This involves the transfer of a proton from one site to another after photoexcitation, producing the corresponding tautomer (Scheme 1). There is a preponderance of ESIPT in a wide variety of molecular systems, ranging from the photophysics of Alizarin^1–3^ (1,2–dihydroxyanthraquinone, one of the oldest dyes known^4^) to the translocation of protons in biological systems.^5–7^ Its ultrafast nature and sensitivity to the local environment have enabled applications ranging from fluorescent sensors to photostabilizers and organic optoelectronics.^8–12^ Thus, much effort has been directed in the past decades toward understanding the dynamics and mechanism of ESIPT in several model systems, such as indoles, quinolines, carboxylic acids, among others.^13–15^ However, a generalized mechanistic consensus on the process remains difficult to establish.^6,16–18^

**Scheme 1:**
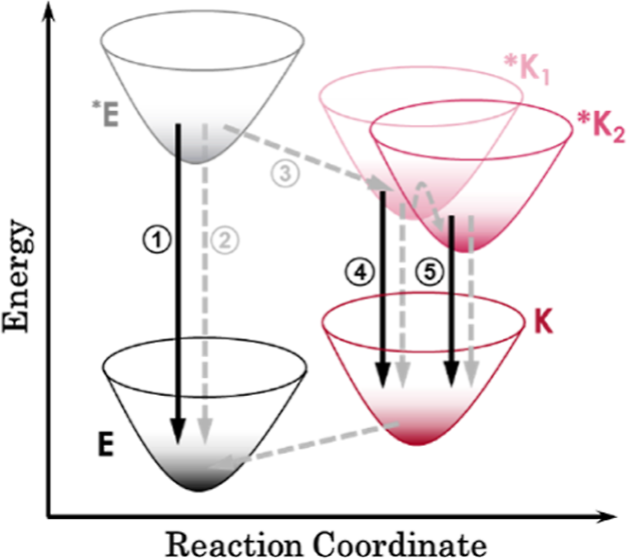
General Qualitative Scheme for ESIPT^a^

a Photoexcitation populates the enol excited state (*E), which may directly decay 1) radiatively (solid black arrow) or 2) non-radiatively (dashed grey arrow) to the ground state, or instead 3) tautomerize to the monoketo (*K1) or diketo (*K2) excited state, with the two processes occurring simultaneously, sequentially, or both. These keto excited states may also decay radiatively or non-radiatively (4 and 5) to their respective keto ground state(s) (K), which eventually relax back to the enol (E).

The superficially simple scheme belies remarkably intricate dynamics: depending on the system chosen, the proton transfer time can range anywhere from sub-100 fs^19^ to several picoseconds^20^—sometimes within the same molecule! ^6,7,21^ Extrinsic factors such as hydrogen bonding strength, pH, and solvent polarity play into the photophysics, in addition to, of course, the molecule’s intrinsic peculiarities which shape the excited state potential energy surface (PES). These intrinsic factors include, but are not limited to, the rigidity or flexibility of the molecular scaffold, its structure, the number and arrangement of proton donor and acceptor groups, and their spatial proximity. For instance, in cases where the molecule features two proton transfer sites, questions emerge regarding the sequential or concerted nature of the proton transfer(s), their rates, and identity of the resulting products: in one model system, an anomalous isotope effect has been shown.^22^ The amount of vibrational excess energy delivered by photoexcitation has been demonstrated to influence product yields as well.^21^ Nevertheless, the vast majority of extant studies have focused primarily on excitation of the lowest electronic state (S_1_).

In this work, we probe ESIPT dynamics in salicylaldehyde azine (SAA) and 1,5–dihydroxyanthraquinone (DHAQ) using ultrafast transient absorption (TA) spectroscopy, with sub-30 fs resolution in the critical UV spectral range. Both molecules in question (Figure 1a) present cases where double proton transfer is possible in principle; which is to say that both mono or diketo products may be formed pursuant to photoexcitation. Both offer a platform for exploring multisite ESIPT and its sensitivity to excitation wavelength.

**Figure 1:**
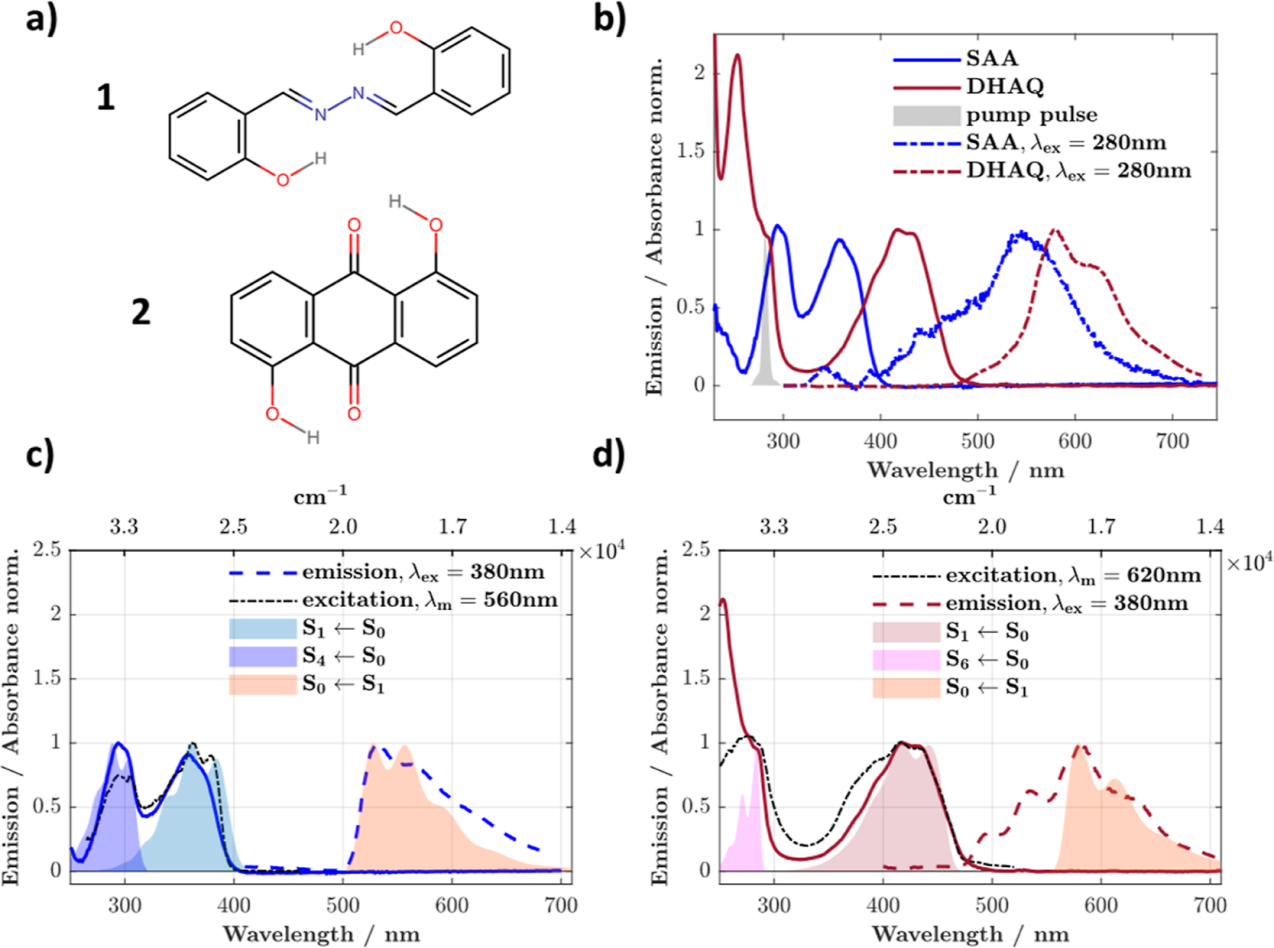
(a) Structures of the molecules under study. (b) Peak normalized steady-state absorption and emission spectra for SAA and DHAQ in THF. Dotted blue and dotted maroon lines show emission when exciting at 280 nm; this is the S_4_ ← S_0_ transition for SAA and S_6_ ← S_0_ transition for DHAQ, confirmed from TD-DFT and well matched FCHT progressions. Peak normalized emission and excitation spectra recorded at 77 K in glassy 2-MeTHF for (c) SAA and (d) DHAQ (λ_ex_ = 380 nm). Room temperature absorption in solid blue (SAA) and maroon (DHAQ) in all panels. Computed absorption spectra for the indicated transitions are overlaid, together with the emission(s) from the monoketo tautomer (shaded areas) in (c) and (d). Note that spectra have been shifted to match the experimental data to account for the small energetic shifts in transitions typically observed in TD-DFT.

While some ultrafast experimental studies exist on DHAQ in the literature,^23–25^ these were limited to a time resolution of 200–300 fs, precluding direct observation of the ESIPT process. The precise rate and the nature of product(s) formed are therefore not known—published experimental and theoretical work^26–28^ suggests that both mono and diketo products may be formed. Thus, this investigation presents the first direct ultrafast observation of ESIPT in DHAQ on a sub-100 fs time scale, revealing significantly faster proton-transfer dynamics in this rigid polyaromatic framework. In contrast, for SAA, an ultrafast time-resolved fluorescence (TRF) study has been executed by Joo and co-workers,^29^ in addition to other TA studies on longer time scales,^30,31^ all focused on S_1_ excitation. In the former, with a ∼50 fs excitation pulse, the rise time of the product emission was tracked to extract an ESIPT time constant of ∼22 ± 5 fs. The experimental coherent vibrational spectrum (CVS) was also compared to computational data to assert the major product was the monoketo isomer. While TRF offers the benefit of selectively probing the excited state surface, with TA measurements made herein, there is the advantage of better time resolution, additional spectral information and a more complete description of the photophysical behavior.

Excitation into the higher energy electronic transitions in these systems presents an informative contrast to examine the impact of excess excitation and vibrational energy on the mechanism and time scale of ESIPT, in addition to the influence of molecular architecture. The high temporal resolution also allows for the study of the influence on the observed coherent oscillations (e.g., due to the presence of conical intersections). On the one hand, the observation of coherent nuclear wavepackets accompanying ESIPT have been furnished as evidence for skeletal mode assistance.^19,32^ On the other hand, they have been argued to be impulsively generated in the excited state,^29,33^ rendering them independent of the reaction coordinate. Data obtained upon excitation into the S_*n*_ manifold should help shed further insight into their diagnostic relevance.

We find that ESIPT, upon excitation in the S_*n*_ manifold, while remaining ballistic (sub-100 fs) in both cases—exhibiting nominally similar dynamics to those observed previously upon S_1_ excitation—does present interesting differences in the expected and observed coherent oscillations accompanying the reaction in the case of SAA.

## Methods

Samples of SAA and DHAQ were obtained from Santa Cruz Biotechnology, Inc. and used without further purification. The solvents, tetrahydrofuran (THF, spectroscopic grade, Uvasol) and 2-methyl tetrahydrofuran (2-MeTHF, anhydrous, >99% Product No. 414247) were obtained from Sigma-Aldrich (Merck).

Steady-state absorption spectra at room temperature were recorded on a Varian Cary 60 spectrophotometer in 1 × 1 or 1 × 0.1 cm quartz cuvettes. Emission and excitation spectra were recorded on a spectrofluorometer (PTI Quantamaster, Horiba) in 1 × 1 cm quartz cuvettes with a right-angle detection geometry. For 77 K measurements, a coldfinger dewar apparatus made from Suprasil quartz was used; samples were made in EPR tubes and liquid nitrogen was used as coolant. Details pertaining to slit-widths and integration times are provided in the Supporting Information.

For longer time scale (0.5 ps–5 ns) TA measurements, ultrafast pulses were generated from a Ti:sapphire regenerative amplifier system (Coherent Astrella, 800 nm, 45 fs, 1 kHz).^34^ Part of the output was directed to a computer-controlled commercial optical parametric amplifier (Coherent, OPerA Solo) to generate the required pump wavelength, while the other was utilized to generate a supercontinuum probe (330 nm–640 nm) by focusing on a CaF_2_ crystal. Different time points were collected by delaying the probe with respect to the pump, for which an automated mechanical delay stage was used. A mechanical chopper blocked every other pump pulse, and difference between spectra recorded with “pump on” and “pump off” furnished the Δ*A* spectrum. Measurements were carried out at magic angle, and pump–probe overlap was optimized at the sample (1 mm thick quartz cuvette). A portion of the probe was reflected before the sample stage to generate a reference spectrum; the latter was compared with the signal to keep fluctuations <5%. Pump power was recorded using an optical sensor, and five scans were averaged for each measurement, with an integration time of 1s at each delay. For short time scale broadband TA measurements, a custom built noncollinear optical parametric amplifier (NOPA) was used instead to generate the UV pulses, in a setup previously described.^35,36^ Briefly, the output from a Coherent Libra (800 nm, 100 fs, 1 kHz) was frequency doubled and noncollinearly mixed with a white-light continuum generated in a 2 mm thick sapphire plate within a 1 mm thick β-barium borate (BBO) crystal, yielding broadband pulses in the visible. These pulses were compressed to transform-limited duration using chirped dielectric mirrors, then frequency-doubled in a 20 μm-thick BBO, resulting in broadband UV pump pulses that could be tuned within the range of 250–300 nm. The pulses were characterized by two-dimensional spectral interferometry and further compressed to 24 fs (fwhm) using a MgF_2_ prism pair, with an overall temporal resolution estimated to be < 30 fs.^37^ The pump pulses were tuned to 282 nm for the experiment, and probe pulses were generated in a CaF_2_ plate by focusing part of the 800 nm output. Pump and probe beams were noncollinearly overlapped at the sample (1 mm quartz cuvette), and measurements were made at the magic angle (54.7°) to avoid polarization effects. To prevent photodegradation, the sample was continuously circulated through the measurement region using a 5 mL stock solution. The steady-state absorption spectra recorded before and after the TA experiments confirmed the absence of photodamage.

All calculations were performed at the B3LYP-D3/6–311G­(d,p) level of theory,^38–44^ with a polarizable continuum model (PCM) model^45–47^ to account for solvent effects. Dichloromethane (DCM) and THF were used as solvents for SAA and DHAQ, respectively. The ground and excited state geometry optimizations were carried out using density functional theory (DFT) and time dependent DFT (TD-DFT). Geometry optimization to the respective electronic states was confirmed via lack of imaginary frequencies in the subsequent frequency calculations. The vibrational reorganization energies were calculated using the ground state normal mode basis. All calculations were carried out using the Gaussian 16 program package.^48^

## Results and Discussion

### Steady-State Spectroscopy

Peak normalized absorption and emission data for SAA and DHAQ can be seen in Figure 1b. The relevant electronic transitions were also computed using DFT and TD-DFT, invoking Franck–Condon-Herzberg–Teller (FCHT) analysis and are overlaid on the low temperature (77 K) experimental data in Figure 1c,d. Excitation into the lowest energy absorption band (i.e., S_1_ excitation) in both molecules, i.e. 380 nm in SAA and 420 nm in DHAQ, results in a red-shifted emission, peaking at 560 and 580 nm for SAA and DHAQ, respectively (Stokes shifts of 9920 and 6682 cm^–1^). This is characteristic of ESIPT due to population of the keto tautomer(s), in good agreement with previous reports in the literature,^49,50^ as well as monoketo tautomer energies and emission spectra computed herein (Table S1, Figure 1c,d).

The emission quantum yield for DHAQ could be determined as ∼1.9% using [Ru­(bpy)_3_]^2+^ as standard (Figure S2), while that for SAA was an order of magnitude lower, ca. 0.17% (Figure S2). For DHAQ, the intersection of the absorption and emission spectra at 480 nm (∼2.58 eV) provides a first estimate of the 0–0 energy. At 77 K (Figure 1c,d), the bands develop vibronic structure, characterized by a progression of ca. 1200 and 1435 cm^–1^ for SAA and DHAQ, respectively. Computations suggest that the observed frequencies may be attributed to C=C stretching modes (Tables S2 and S3).

Excitation at 280 nm into higher energy electronic transitions precipitates notably different emission behavior. For SAA, the room temperature emission peaks in the blue and becomes broader, owing in part to solvent contributions, obscuring the red-shifted emission. The low emission quantum yields (Figure S2 and associated text) coupled with solvent background preclude the collection of reliable excitation spectra. At 77 K, on the other hand, the red emission is recovered and is identical to that observed upon S_1_ excitation, albeit with lowered yield, as indicated by the excitation spectra. Taken together with the computed FCHT vibronic spectra (Figure S5), the observed blue emission is tentatively assigned to the enol-tautomer.

For DHAQ, the trend is reversed, and excitation into the S_6_ transition results in qualitative reproduction of the keto tautomer emission at room temperature, albeit with a lowered quantum yield of 1.2% (Figure S2), revealing the presence of additional decay pathways upon excitation at this wavelength. A blue emission from 420 to 520 nm is only resolved clearly in the low temperature data (Figure S2), with the excitation spectrum indicating anti-Kasha emission largely sourced in higher-lying excited states, and possible minor contribution(s) from the lowest-lying enol excited state (Figure S5). This information will assist in interpretation of the ultrafast TA data; key information is compiled in Table 1 for ready reference.

**Table 1:** Room Temperature Emission Data for SAA and DHAQ

	λ_em_/nm (ϕ_em_ – S_1_)	λ_em_/nm (ϕ_em_ – S_*n*_)
SAA	560 (0.0017)	545^a^ (0.0039)
DHAQ	580 (0.019)	580 (0.012)

a After correcting for solvent background, see Supporting Information.

### Time-Resolved Spectroscopy

Excitation into the S_4_ transition at 282 nm in SAA results in clear observation of the ESIPT process on sub-100 fs time scales, Figure 2a. The spectral evolution is characterized by growth of stimulated emission (SE) in the red (>550 nm) expected from the formation of the monoketo product. A concomitant increase in the excited state absorption (ESA) at ca. 410 nm is also observed, together with band-narrowing and spectral-blue shifting of the ESA peak initially at 500 to 480 nm. These initial changes are complete within 300 fs. Taken in conjunction with the steady-state and computational emission data, the spectral changes are consistent with loss of SE from a higher-lying enol excited state between 400–500 nm, where at least part of it undergoes ESIPT competitively with other decay channels. Global analysis (Figures 2c and S3) of the data set allows for extraction of a time-constant of ∼30 ± 5 fs, and the associated decay associated spectrum (DAS1) is overlaid in Figure 2a, where the SE component is evident below 520 nm. The observed time constant is similar to the one obtained upon S_1_ excitation previously by Joo and co-workers. An additional ∼570 fs component is observed in the short-time scale data, corresponding to a ∼2 ps component in the long-time scale measurements (DAS, Figure S4); both are assigned to intramolecular vibrational redistribution (IVR) and solvation-driven relaxation of the keto excited state, manifested primarily as a gradual redistribution of ESA intensity between the 400 and 500 nm bands toward the final spectral shape.

**Figure 2:**
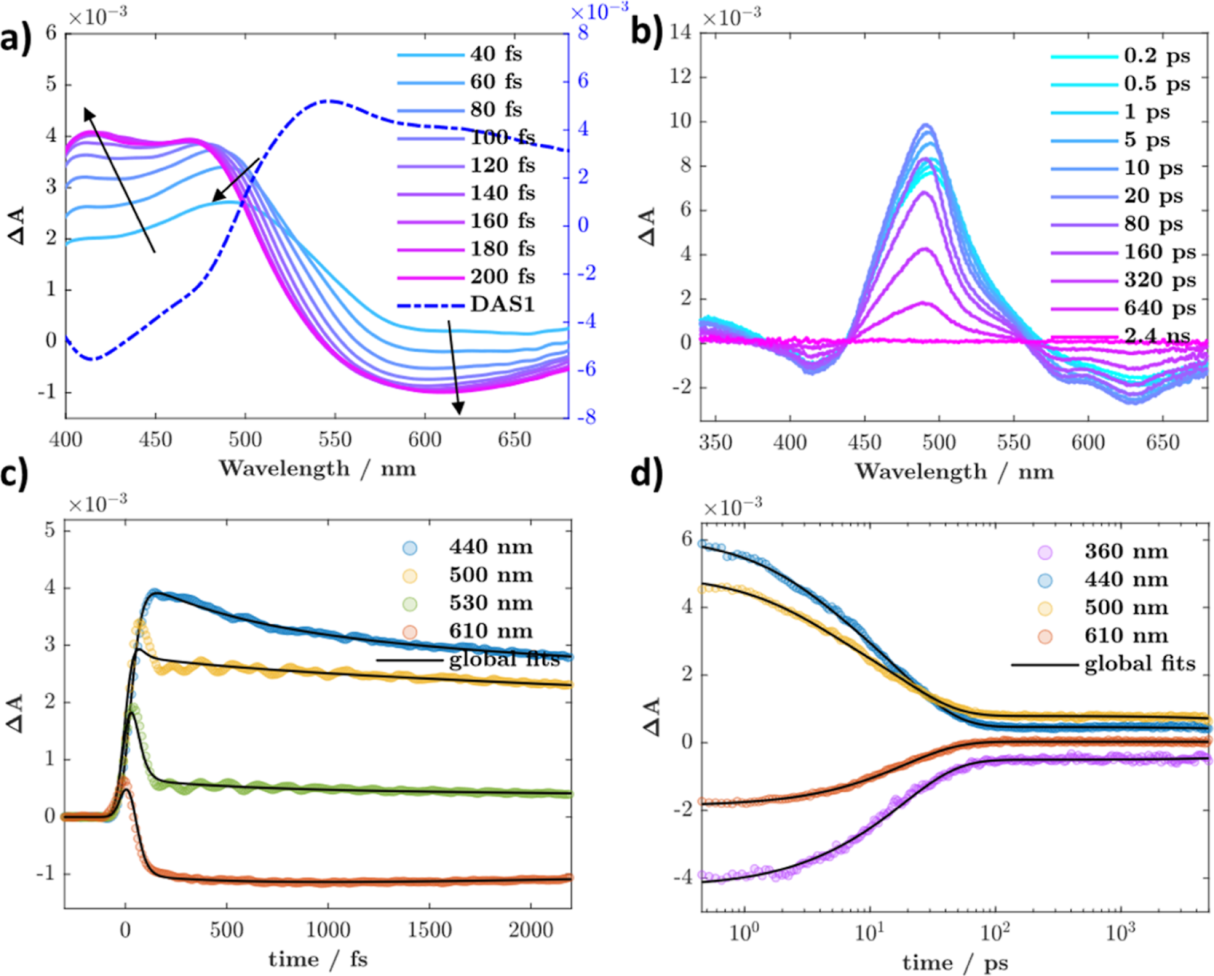
(a) Early time scale femtosecond TA data recorded for SAA in THF (λ_ex_ = 282 nm, excitation fluence = 235 μJ/cm^2^, absorption ≈2.1 at the excitation wavelength), arrows guide spectral evolution. “DAS1” is the decay associated spectrum associated with the ESIPT process. (b) Late time scale femtosecond TA data recorded for SAA in THF (λ_ex_ = 280 nm, excitation fluence = 330 μJ/cm^2^, absorption ≈0.31 at the excitation wavelength). (c) Kinetics monitored at indicated wavelengths from (a), together with global fits. (d) Kinetics monitored at indicated wavelengths from (b), together with global fits.

The long time scale data (Figure 2b) tracks the fate of the excited monoketo tautomer, which proceeds to decay (Figure 2d) with a time constant of ∼18 ps (global analysis, Figure S3), in good agreement with previous reports for S_1_ excitation.^49,51^ The majority of the excited tautomer population decays directly to a vibronically excited keto geometry on the ground state PES. The ESA features on longer time scales observed upon S_1_ excitation are narrower as opposed to S_4_ (Figure S3). Furthermore, an initial isosbestic point is observed at ca. 556 nm upon S_4_ excitation, which proceeds to shift to ca. 525 nm, while the shift is less pronounced (532 to 520 nm) when exciting the S_1_ state. These observed differences may be ascribed to the population of a hotter product state upon S_4_ excitation, i.e. with greater vibrational energy excess. Ground state recovery is not complete in the time-window of the experiment (>2 ns). The minor long-lived component can be assigned to a twisted photochromic conformation of the keto isomer based on previous reports.^30^

Finally, coherent oscillations are also observed upon S_4_ excitation, as has been reported for S_1_ excitation using TRF. The frequencies obtained from fast Fourier transform (FFT) are presented in Figure 3a, the so-called experimental CVS. Comparison with the solvent response (Figure 3c) under identical conditions suggests all observed dominant frequencies (intensity >1.5) find their origins in the molecule of interest. Curiously, higher energy excitation results in only a single mode with frequency 235 cm^–1^ in the region >500 nm, where SE may be expected to dominate. The dominant mode in the ESA region is ∼130 cm^–1^, however, with a less intense lower frequency mode at ∼55 cm^–1^. Given the onset of SAA absorption at wavelengths shorter than 400 nm, both SE and ESA regions in this case may potentially report on excited-state vibrational wavepackets. The observed modes are in good agreement with those seen in the experimental CVS (intense peaks at 134 and 233 cm^–1^) of the aforementioned study—barring the absence of the 134 cm^–1^ mode in the SE region. It is important to note that direct comparison of coherences observed in transient absorption and time-resolved fluorescence is not straightforward, as the TA signal arises from the superposition of ESA and SE contributions with different phases in the >500 nm region. Phase cancellation^52^ can therefore reduce the apparent amplitude of low-frequency modes (55 and 130 cm^–1^) that are clearly observed in the pure ESA region. While this effect may contribute to the suppression of low-frequency features in the SE-dominated window, it cannot be uniquely disentangled from changes in excited-state potential energy surfaces induced by higher-energy excitation.

**Figure 3:**
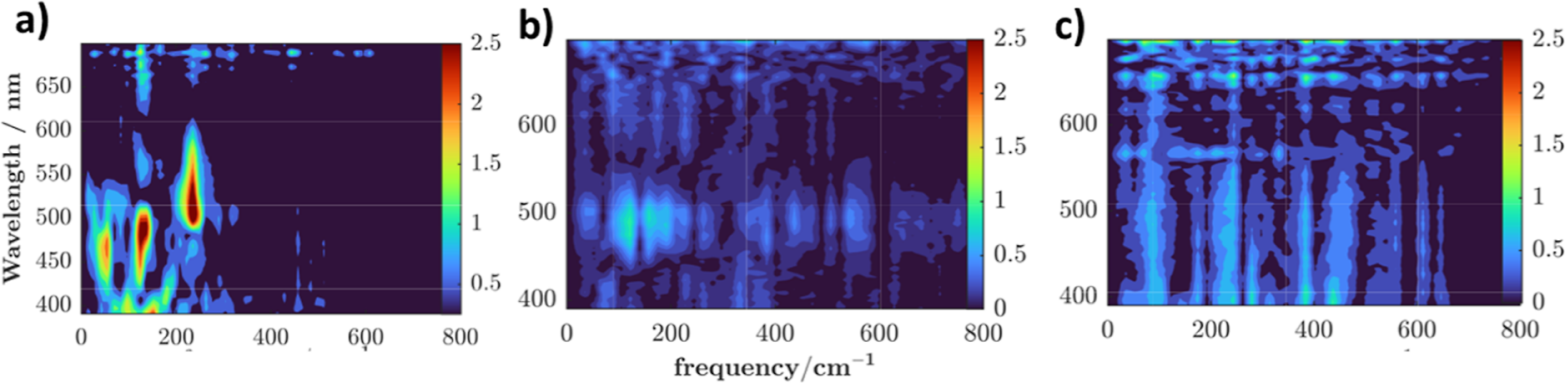
Fast Fourier transform (FFT) maps of the residuals obtained after the subtraction of population dynamics from the TA data (triexponential global fit, see Supporting Information for details). (a) SAA, (b) DHAQ, and (c) THF (solvent only). The plotted intensities are Fourier amplitudes.

To gain further insight into the origins of the differences in the observed CVS, reorganization energies were computed for excitation into the S_1_ and S_4_ manifolds, and can be seen in Figure 4a. When moving from S_1_ to S_4_, a clear attenuation in the intensity of the mode at ∼130 cm^–1^ can be seen, in addition to a more pronounced magnitude for the 235 cm^–1^ vibration, owing to a change in the potential energy landscape. Specifically, the variations may be interpreted as a direct consequence of changes in mode specific equilibrium displacements that are reflected in the magnitude of the reorganization energies. A salient feature of these modes is that they are symmetric vibrations which modulate the N–H distance (Figure S6), potentially implicating them in ESIPT. A more subtle—sometimes overlooked—feature is that these modes are present in both the reactant (enol) and product (keto) state, albeit with minor frequency shifts associated with the changed geometry (Figure S8). In the present case, all modes are present in both the reactant and product basis, barring the one at 130 cm^–1^, which is exclusive to the reactant state. Thus, the observed vibrations are neither impulsively generated, nor exclusive to the product state. In fact, they are better interpreted as modes which originate in the reactant state as a consequence of broadband excitation, which are then probed in the product state following the reaction.

**Figure 4:**
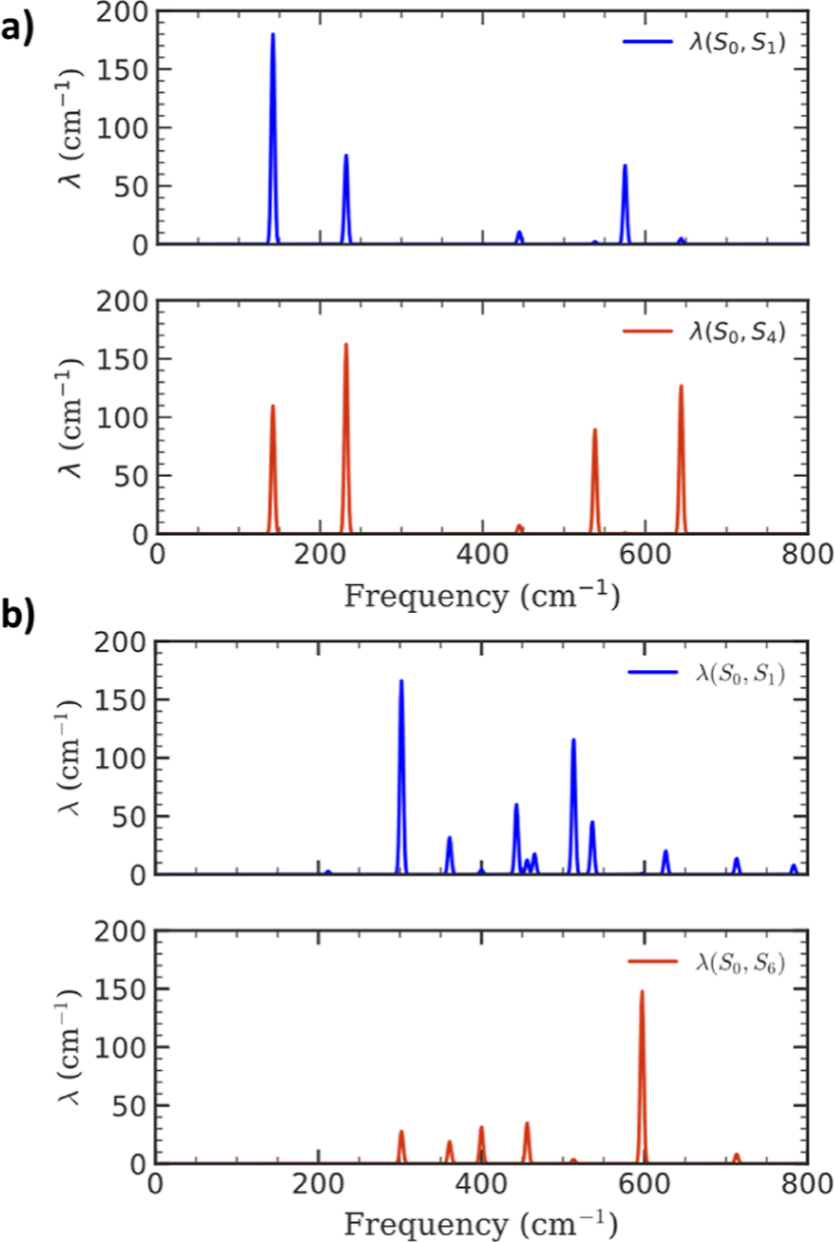
Computed reorganization energies for S_1_ (blue) and S_*n*_ (red) excitation. (a) SAA and (b) DHAQ.

In the previous study, the presence of a mode at 322 cm^–1^ (subsequent to a linear prediction from singular value decomposition analysis, which helps improve the signal-to-noise of the experimental CVS) was used as diagnostic for the population of the monoketo isomer by comparison of the experimental and calculated CVS of various possible products. Inasmuch as the final state populated here is also the same as that after S_1_ excitation—given the identical long time scale lifetimes of the product—the diagnostic role of the observed modes and comparative CVS for product assignment is not general. The possibility that observed coherent oscillations are involved in the reaction coordinate—so-called skeletal mode assistance—is an open question.

For DHAQ, excitation at 282 nm should populate primarily the S_6_ manifold, and the resulting early time scale dynamics are plotted in Figure 5a. A clear growth in the ESA and SE can be seen, peaking at 494 and 631 nm, respectively, indicating formation of the keto isomer, as product emission can be expected at wavelengths >500 nm based on the steady-state data. The presence of relatively unperturbed isosbestic points at 530 and 450 nm points to the conversion of one state to another, with population of other states—if present—being a minor component. Global analysis of the data allows for the determination of an ultrafast ESIPT time constant of 85 ± 5 fs (Figure S4). This is shorter than previously reported time constants by a factor of ca. 2.5, where the rise of the emission from the keto isomer was not fully resolved.^23–25^ This longer observed lifetime of the enol isomer compared to SAA may be rationalized by the constrained nuclear motion upon photoexcitation, as reflected in the calculated coherent vibrational spectrum and reorganization energies seen in Figure 4 (red), reducing the contribution of vibrationally assisted (passive) ESIPT. A proper characterization of the mechanism, however, would require further quantum dynamics studies. Another component is also present, which is fully resolved in the global analysis of the longer time scale data as ∼9 ps (Figure 5b). This is followed by ground state recovery in ∼350 ps (Figure 5d). The longer observed lifetime of the keto isomer for DHAQ is consistent with its rigid structure and thus lowered rate of nonradiative decay.

**Figure 5:**
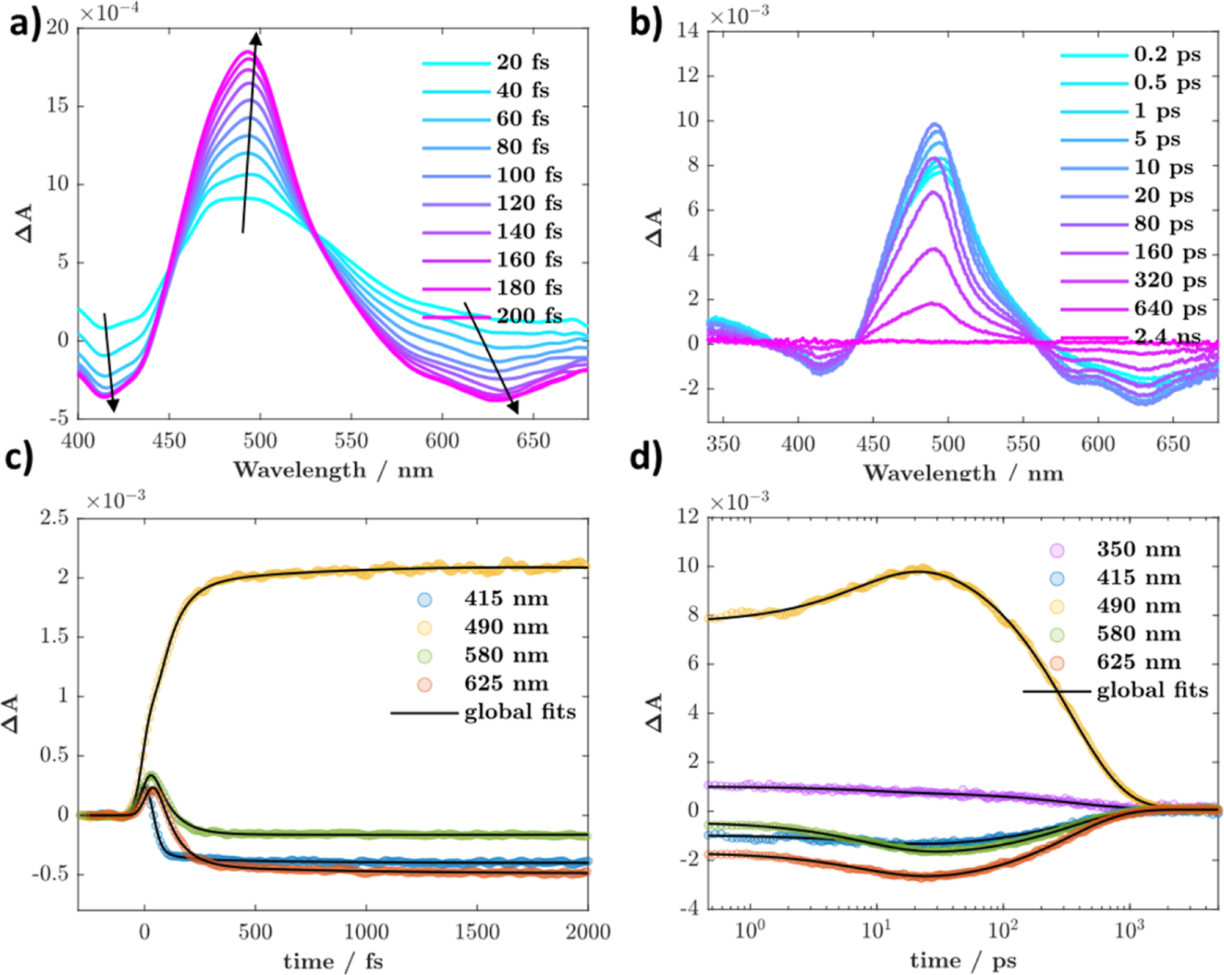
(a) Early time scale femtosecond TA data recorded for DHAQ in THF (λ_ex_ = 282 nm, excitation fluence = 235 μJ/cm^2^, absorption ≈2.5 at the excitation wavelength), arrows guide spectral evolution. (b) Late time scale femtosecond TA data recorded for DHAQ in THF (λ_ex_ = 280 nm, excitation fluence = 330 μJ/cm^2^, absorption ≈0.59 at the excitation wavelength). (c) Kinetics monitored at indicated wavelengths from a), together with global fits. (d) Kinetics monitored at indicated wavelengths from (b), together with global fits.

The assignment of the 9 ps component is not straightforward; it is dependent on the excitation wavelength, increasing to ∼11 ps upon 380 nm excitation (Figure S4). In the simplest case, the observed band narrowing for the ESA and greater structure in the SE band can be assigned to vibrational cooling. On the other hand, the continued growth in ESA and SE may well be interpreted as a second, slower, sequential proton transfer event occurring from the still thermalizing monoketo product formed in <100 fs. This would make the case akin to that asserted for model double proton transfer systems such as [2,2′-bipyridyl]-3,3′-diol and the 7-azaindole dimer,^6,53^ wherein ballistic ESIPT is observed producing both mono- and diketo isomers on sub-100 fs time scales, followed by the monoketo intermediate converting to the diketo on a time scale of several picoseconds. The latter view has been challenged for the case of the 7-azaindole dimer.^17,54^ Nevertheless, it has been shown in other cases that the amount of monoketo intermediate formed is contingent on the excess (vibrational) excitation energy.^21^

The relative change in ESA peak amplitude on long time scales is approximately 30% on 280 nm excitation as opposed to 15% on 380 nm excitation; a degree of IVR can be assumed in any case. Theory posits the monoketo isomer as the lowest-lying excited state (Table S1); the diketo isomer also presents a local minimum and is only 0.15 eV higher in energy,^27^ and could be populated upon S_6_ excitation. Determination of the product identity will therefore require additional work. Greater aromatic stability of the monoketo isomer further corroborates the computational results, suggesting it is the likely dominant product.^55^ Finally, the residual FFT map for DHAQ is presented in Figure 3b. The peak intensities are on the same scale as the solvent response (Figure 3c), and the pattern >550 nm is similar, suggesting that no nuclear wavepackets exclusive to the molecule’s excited state are being observed. A minor response is observed in the ESA region around 490 nm, with frequencies 122 and 157 cm^–1^. As can be seen in Figure 4b, in contrast to SAA, the computed λ_i_ for low frequency modes in DHAQ are mostly low for the S_6_ state, which explains the less pronounced peak intensities in the CVS of DHAQ. The mode at ∼600 cm^–1^ presents a notable exception, but is nevertheless not observed in experiment. In general, vibrations behaving as promoting modes can have faster dephasing times,^56^ rendering them unobservable—which may well be the case here. Other causes may not be ruled out, however. Ultimately, these results highlight the fact that while experimental observation of coherences does not necessarily implicate them in the reaction, nonobservance also does not preclude their involvement. Each case demands careful comparison of experiment and theory to accurately delineate their mechanistic role.^57^

## Conclusions

To summarize, ESIPT upon ultraviolet excitation was investigated in two model compounds, SAA and DHAQ, using transient absorption spectroscopy with sub-30 fs resolution, allowing for direct observation of the proton transfer process on sub-100 fs time scales. Ultraviolet excitation at 282 nm was employed to investigate how higher-lying electronically excited states in particular modulate the ensuing cascade of photophysical processes. In the case of SAA, it was found that, while the time scale of ESIPT (∼30 fs) remained essentially invariant between S_1_ and S_4_ excitation, there was a reduction in yield of the monoketo tautomer due to presence of additional decay pathways from higher-lying states. Importantly, the observed coherent oscillations associated with the reaction changed upon S_4_ excitation. Comparison with data obtained on lower-energy excitation suggests that (a) not all observed nuclear wavepackets are impulsively generated as a result of the reaction, and (b) an unequivocal CVS may not exist for a given product state.

For DHAQ, the first direct observation of the ESIPT time constant was made, which could be determined as ∼85 fs. The near 3-fold reduction in rate compared to SAA is consistent with the higher structural rigidity of DHAQ compared to the former, although other factors, such as donor–acceptor bond distances, can also be expected to be critical for rate determination.^58^ A second, longer ∼9 ps component was also observed in the transient data, which can potentially be assigned to a second proton transfer process in tandem with vibration cooling. The steady-state and time-resolved spectral data, together with the results from TD-DFT calculations, suggest however that the dominant product for photoinduced ESIPT in DHAQ is the monoketo isomer. Taken together, the results not only provide first experimental data for ESIPT on S_*n*_ excitation in these two model systems, but also provide a critical perspective on the diagnostic relevance of coherent oscillations which oftentimes accompany this class of reactions.

## Supplementary Material



## Notes

Experimental setup details, additional steady state spectroscopic data, additional fs-TA data and global analysis, quantum yield determination, fitting procedures, additional computational data (PDF)

## References

[1] Jen M., Lee S., Jeon K., Hussain S., Pang Y. (2017). Ultrafast Intramolecular
Proton Transfer of Alizarin Investigated by Femtosecond Stimulated
Raman Spectroscopy. J. Phys. Chem. B.

[2] Jen M., Jeon K., Lee S., Hwang S., Chung W., Pang Y. (2019). Ultrafast Intramolecular
Proton Transfer Reactions and Solvation
Dynamics of DMSO. Struct. Dyn..

[3] Lee S., Lee J., Pang Y. (2015). Excited State Intramolecular Proton Transfer of 1,2-Dihydroxyanthraquinone
by Femtosecond Transient Absorption Spectroscopy. Curr. Appl. Phys..

[4] De
Santis D., Moresi M. (2007). Production of Alizarin Extracts from
Rubia Tinctorum and Assessment of Their Dyeing Properties. Ind. Crops Prod..

[5] Chai S., Zhao G.-J., Song P., Yang S.-Q., Liu J.-Y., Han K.-L. (2009). Reconsideration of the Excited-State
Double Proton
Transfer (ESDPT) in 2-Aminopyridine/Acid Systems: Role of the Intermolecular
Hydrogen Bonding in Excited States. Phys. Chem.
Chem. Phys..

[6] Douhal A., Kim S. K., Zewail A. H. (1995). Femtosecond
Molecular Dynamics of
Tautomerization in Model Base Pairs. Nat..

[7] Douhal A., Lahmani F., Zewail A. H. (1996). Proton-Transfer
Reaction Dynamics. Chem. Phys..

[8] Sedgwick A. C., Wu L., Han H. H., Bull S. D., He X. P., James T. D., Sessler J. L., Tang B. Z., Tian H., Yoon J. (2018). Excited-State
Intramolecular Proton-Transfer (ESIPT) Based Fluorescence Sensors
and Imaging Agents. Chem. Soc. Rev..

[9] Kwon J. E., Park S. Y. (2011). Advanced Organic
Optoelectronic Materials: Harnessing
Excited-State Intramolecular Proton Transfer (ESIPT) Process. Adv. Mater..

[10] Gu H., Wang W., Wu W., Wang M., Liu Y., Jiao Y., Wang F., Wang F., Chen X. (2023). Excited-State
Intramolecular Proton Transfer (ESIPT)-Based Fluorescent Probes for
Biomarker Detection: Design, Mechanism, and Application. Chem. Commun..

[11] Tang K.-C., Chang M.-J., Lin T.-Y., Pan H.-A., Fang T.-C., Chen K.-Y., Hung W.-Y., Hsu Y.-H., Chou P.-T. (2011). Fine Tuning
the Energetics of Excited-State Intramolecular Proton Transfer (ESIPT):
White Light Generation in A Single ESIPT System. J. Am. Chem. Soc..

[12] Manolova Y., Marciniak H., Tschierlei S., Fennel F., Kamounah F. S., Lochbrunner S., Antonov L. (2017). Solvent Control of Intramolecular
Proton Transfer: Is 4-Hydroxy-3-(Piperidin-1-Ylmethyl)-1-Naphthaldehyde
a Proton Crane?. Phys. Chem. Chem. Phys..

[13] Joshi H. C., Antonov L. (2021). Excited-State Intramolecular
Proton Transfer: A Short
Introductory Review. Molecules.

[14] Reimann L. K., Dalberto B. T., Schneider P. H., de Castro Silva Junior H., Rodembusch F. S. (2025). Benzazole-Based
ESIPT Fluorophores: Proton Transfer
from the Chalcogen Perspective. A Combined Theoretical and Experimental
Study. J. Fluoresc..

[15] Mishra V. R., Ghanavatkar C. W., Sekar N. (2020). Towards NIR-Active Hydroxybenzazole
(HBX)-Based ESIPT Motifs: A Recent Research Trend. ChemistrySelect.

[16] Alarcos N., Gutierrez M., Liras M., Sánchez F., Douhal A. (2015). An Abnormally Slow Proton Transfer Reaction in a Simple
HBO Derivative Due to Ultrafast Intramolecular-Charge Transfer Events. Phys. Chem. Chem. Phys..

[17] Takeuchi S., Tahara T. (2007). The Answer to Concerted versus Step-Wise Controversy
for the Double Proton Transfer Mechanism of 7-Azaindole Dimer in Solution. Proc. Natl. Acad. Sci. U. S. A..

[18] Zhou P., Han K. (2018). Unraveling the Detailed Mechanism
of Excited-State Proton Transfer. Acc. Chem.
Res..

[19] Schriever C., Barbatti M., Stock K., Aquino A. J. A., Tunega D., Lochbrunner S., Riedle E., de Vivie-Riedle R., Lischka H. (2008). The Interplay of Skeletal
Deformations and Ultrafast
Excited-State Intramolecular Proton Transfer: Experimental and Theoretical
Investigation of 10-Hydroxybenzo­[h]­Quinoline. Chem. Phys..

[20] Share P., Pereira M., Sarisky M., Repinec S., Hochstrasser R. M. (1991). Dynamics
of Proton Transfer in 7-Azaindole. J. Lumin..

[21] Marks D., Prosposito P., Zhang H., Glasbeek M. (1998). Femtosecond Laser Selective
Intramolecular Double-Proton Transfer in [2,2′-Bipyridyl]-3,3′-Diol. Chem. Phys. Lett..

[22] Zhang X., Schwarz K. N., Zhang L., Fassioli F., Fu B., Nguyen L. Q., Knowles R. R., Scholes G. D. (2022). Interference of
Nuclear Wavepackets in a Pair of Proton Transfer Reactions. Proc. Natl. Acad. Sci. U. S. A..

[23] Sun C., Li Y., Li B., Han J., Zhou Q., Yin H., Shi Y. (2020). Ingeniously Regulating the Antioxidant Activities of
Hydroxyanthraquinone-Based
Compounds via ESIPT Reaction: Combining Experiment and Theory Methods. J. Mol. Liq..

[24] Wang Z.-R., Zhu L.-X., Zhang X.-L., Li B., Liu Y.-L., Wan Y.-F., Li Q., Wan Y., Yin H., Shi Y. (2022). Effect of Solvent Polarity on Excited-State Double
Proton Transfer
Process of 1,5-Dihydroxyanthraquinone. Chin.
J. Chem. Phys..

[25] Hwang H., Tiwari V., Duan H.-G., Bittmann S. F., Tellkamp F., Jha A., Miller R. J. D. (2022). Two-Dimensional
Confinement for Generating Thin Single
Crystals for Applications in Time-Resolved Electron Diffraction and
Spectroscopy: An Intramolecular Proton Transfer Study. Chem. Commun..

[26] Flom S. R., Barbara P. F. (1985). Proton Transfer and Hydrogen Bonding in the Internal
Conversion of S1 Anthraquinones. J. Phys. Chem..

[27] Zheng D., Zhang M., Zhao G. (2017). Combined TDDFT and
AIM Insights into
Photoinduced Excited State Intramolecular Proton Transfer (ESIPT)
Mechanism in Hydroxyl- and Amino-Anthraquinone Solution. Sci. Rep..

[28] Zhou Q., Du C., Yang L., Zhao M., Dai Y., Song P. (2017). Mechanism
for the Excited-State Multiple Proton Transfer Process of Dihydroxyanthraquinone
Chromophores. J. Phys. Chem. A.

[29] Lee S. N., Ahn J., Joo T. (2022). Coherent Vibrational
Spectrum via Time-Resolved Fluorescence
for Molecular Dynamics and Identification of Emitting Species-Application
to Excited-State Intramolecular Proton Transfer. J. Phys. Chem. A.

[30] Ziółek M., Burdziński G., Douhal A. (2012). Long-Living Structures
of Photochromic
Salicylaldehyde Azine: Polarity and Viscosity Effects from Nanoseconds
to Hours. Photochem. Photobiol. Sci..

[31] Ziółek M., Gil M., Organero J. A., Douhal A. (2010). What Is the Difference between the
Dynamics of Anion- and Keto-Type of Photochromic Salicylaldehyde Azine?. Phys. Chem. Chem. Phys..

[32] Takeuchi S., Tahara T. (2005). Coherent Nuclear Wavepacket Motions in Ultrafast Excited-State
Intramolecular Proton Transfer: Sub-30-Fs Resolved Pump-Probe Absorption
Spectroscopy of 10-Hydroxybenzo­[h]­Quinoline in Solution. J. Phys. Chem. A.

[33] Kim C. H., Joo T. (2009). Coherent Excited State Intramolecular Proton Transfer Probed by Time-Resolved
Fluorescence. Phys. Chem. Chem. Phys..

[34] Granados D. A., Du Y. E., Andersson S. J., Cirincione-Lynch A., Cui K., Reinhold A., Jeffrey P. D., Scholes G. D., Hammes-Schiffer S., Knowles R. R. (2025). Iridium Polypyridyl
Carboxylates as Excited-State PCET
Catalysts for the Functionalization of Unactivated C–H Bonds. J. Am. Chem. Soc..

[35] Petropoulos V., Martinez-Fernandez L., Uboldi L., Maiuri M., Cerullo G., Balanikas E., Markovitsi D. (2024). Real-Time Observation of Sub-100-Fs
Charge and Energy Transfer Processes in DNA Dinucleotides. Chem. Sci..

[36] Borrego-Varillas R., Ganzer L., Cerullo G., Manzoni C. (2018). Ultraviolet Transient
Absorption Spectrometer with Sub-20-Fs Time Resolution. Appl. Sci..

[37] Borrego-Varillas R., Oriana A., Branchi F., De Silvestri S., Cerullo G., Manzoni C. (2015). Optimized Ancillae
Generation for
Ultra-Broadband Two-Dimensional Spectral-Shearing Interferometry. J. Opt. Soc. Am. B.

[38] Becke A. D. (1993). Density-functional
Thermochemistry. III. The Role of Exact Exchange. J. Chem. Phys..

[39] Lee C., Yang W., Parr R. G. (1988). Development of the Colle-Salvetti
Correlation-Energy Formula into a Functional of the Electron Density. Phys. Rev. B.

[40] Vosko S. H., Wilk L., Nusair M. (1980). Accurate Spin-Dependent Electron
Liquid Correlation Energies for Local Spin Density Calculations: A
Critical Analysis. Can. J. Phys..

[41] Stephens P. J., Devlin F. J., Chabalowski C. F., Frisch M. J. (1994). Ab Initio Calculation
of Vibrational Absorption and Circular Dichroism Spectra Using Density
Functional Force Fields. J. Phys. Chem..

[42] McLean A. D., Chandler G. S. (1980). Contracted Gaussian
Basis Sets for Molecular Calculations.
I. Second Row Atoms, *Z* = 11–18. J. Chem. Phys..

[43] Grimme S., Antony J., Ehrlich S., Krieg H. (2010). A Consistent and Accurate
Ab Initio Parametrization of Density Functional Dispersion Correction
(DFT-D) for the 94 Elements H-Pu. J. Chem. Phys..

[44] Krishnan R., Binkley J. S., Seeger R., Pople J. A. (1980). Self-consistent
Molecular Orbital Methods. XX. A Basis Set for Correlated Wave Functions. J. Chem. Phys..

[45] Pascual-ahuir J. L., Silla E., Tuñon I. (1994). GEPOL: An Improved Description of
Molecular Surfaces. III. A New Algorithm for the Computation of a
Solvent-Excluding Surface. J. Comput. Chem..

[46] Miertuš S., Scrocco E., Tomasi J. (1981). Electrostatic
Interaction of a Solute
with a Continuum. A Direct Utilizaion of AB Initio Molecular Potentials
for the Prevision of Solvent Effects. Chem.
Phys..

[47] Miertus̃ S., Tomasi J. (1982). Approximate Evaluations
of the Electrostatic Free Energy
and Internal Energy Changes in Solution Processes. Chem. Phys..

[48] Frisch, M. J. ; Trucks, G. W. ; Schlegel, H. B. ; Scuseria, G. E. ; Robb, M. A. ; Cheeseman, J. R. ; Scalmani, G. ; Barone, V. ; Petersson, G. A. ; Nakatsuji, H. ; Li, X. ; Caricato, M. ; Marenich, A. V. ; Bloino, J. ; Janesko, B. G. ; Gomperts, R. ; Mennucci, B. ; Hratchian, H. P. ; Ortiz, J. V. ; Izmaylov, A. F. ; Sonnenberg, J. L. ; Williams-Young, D. ; Ding, F. ; Lipparini, F. ; Egidi, F. ; Goings, J. ; Peng, B. ; Petrone, A. ; Henderson, T. ; Ranasinghe, D. ; Zakrzewski, V. G. ; Gao, J. ; Rega, N. ; Zheng, G. ; Liang, W. ; Hada, M. ; Ehara, M. ; Toyota, K. ; Fukuda, R. ; Hasegawa, J. ; Ishida, M. ; Nakajima, T. ; Honda, Y. ; Kitao, O. ; Nakai, H. ; Vreven, T. ; Throssell, K. ; Montgomery, J. A., Jr. ; Peralta, J. E. ; Ogliaro, F. ; Bearpark, M. J. ; Heyd, J. J. ; Brothers, E. N. ; Kudin, K. N. ; Staroverov, V. N. ; Keith, T. A. ; Kobayashi, R. ; Normand, J. ; Raghavachari, K. ; Rendell, A. P. ; Burant, J. C. ; Iyengar, S. S. ; Tomasi, J. ; Cossi, M. ; Millam, J. M. ; Klene, M. ; Adamo, C. ; Cammi, R. ; Ochterski, J. W. ; Martin, R. L. ; Morokuma, K. ; Farkas, O. ; Foresman, J. B. ; Fox, D. J. Gaussian 16. Revision A.03; Gaussian, Inc.: Wallingford, CT, USA, 2016.

[49] Ziółek M., Kubicki J., Maciejewski A., Naskrȩcki R., Grabowska A. (2004). An Ultrafast Excited State Intramolecular
Proton Transfer
(ESPIT) and Photochromism of Salicylideneaniline (SA) and Its “Double”
Analogue Salicylaldehyde Azine (SAA). A Controversial Case. Phys. Chem. Chem. Phys..

[50] Van
Benthem M. H., Gillispie G. D. (1984). Intramolecular Hydrogen Bonding.
4. Dual Fluorescence and Excited-State Proton Transfer in 1,5-Dihydroxyanthraquinone. J. Phys. Chem..

[51] Ziółek M., Filipczak K., Maciejewski A. (2008). Spectroscopic and Photophysical Properties
of Salicylaldehyde Azine (SAA) as a Photochromic Schiff Base Suitable
for Heterogeneous Studies. Chem. Phys. Lett..

[52] Fitzpatrick C., Odhner J. H., Levis R. J. (2020). Spectral
Signatures of Ground- and
Excited-State Wavepacket Interference after Impulsive Excitation. J. Phys. Chem. A.

[53] Zhang H., van der Meulen P., Glasbeek M. (1996). Ultrafast Single and
Double Proton
Transfer in Photo-Excited [2,2′-Bipyridyl]-3,3′-Diol. Chem. Phys. Lett..

[54] Crespo-Otero R., Kungwan N., Barbatti M. (2015). Stepwise Double Excited-State
Proton
Transfer Is Not Possible in 7-Azaindole Dimer. Chem. Sci..

[55] Wu C.-H., Karas L. J., Ottosson H., Wu J. I.-C. (2019). Excited-State
Proton Transfer Relieves Antiaromaticity in Molecules. Proc. Natl. Acad. Sci. U. S. A..

[56] Sahu A., Tiwari V. (2023). Vibrations That Do Not Promote Vibronic
Coupling Can
Dominate Observed Lineshapes in Two-Dimensional Electronic Spectroscopy. J. Phys. Chem. Lett..

[57] Benny A., Wang Y., Scholes G. D. (2025). The Interplay of
Vibrational Coherence
and Photoinduced Ultrafast Chemical Reactions. J. Am. Chem. Soc..

[58] Zhang M.-T., Irebo T., Johansson O., Hammarström L. (2011). Proton-Coupled
Electron Transfer from Tyrosine: A Strong Rate Dependence on Intramolecular
Proton Transfer Distance. J. Am. Chem. Soc..

