# Supplementary material for: Exploring Excited State Proton Transfer Dynamics upon Ultraviolet Excitation
Source: J Phys Chem A. 2026 Mar 11;130(12):2566–74. doi: 10.1021/acs.jpca.5c08622 (PMC13034412; doi:10.1021/acs.jpca.5c08622)
Supplement: Supplementary file 1 [file jp5c08622_si_001.pdf]

Supporting Information:

Exploring Excited State Proton Transfer Dynamics Upon Ultraviolet Excitation

Nidhi Kaul[1], Alfy Benny[1], Vasilis Petropoulos[2], Michał Maj[3], Giulio Cerullo[2], Margherita Maiuri[2]*, Gregory D. Scholes[1*]

[1]Department of Chemistry, Princeton University, Princeton, New Jersey 08544, United States
[2]Dipartimento di Fisica, Politecnico di Milano, Piazza Leonardo da Vinci 32, 20133 Milano, Italy
[3]Department of Chemistry – Ångström Laboratory, Uppsala University, Box 523, SE-75120 Uppsala, Sweden

*Corresponding Authors:

Gregory D. Scholes (gscholes@princeton.edu)

Margherita Maiuri (margherita.maiuri@polimi.it)

Contents

**1.**	**Steady State Spectroscopy**	S3
**2.**	**Transient Absorption Spectroscopy**	S5
**3.**	**Density Functional Theory and Time-Dependent Density Functional Theory**	S7
**4.**	**References**	S9

1. Steady-State Spectroscopy

1.1. Additional Emission Data

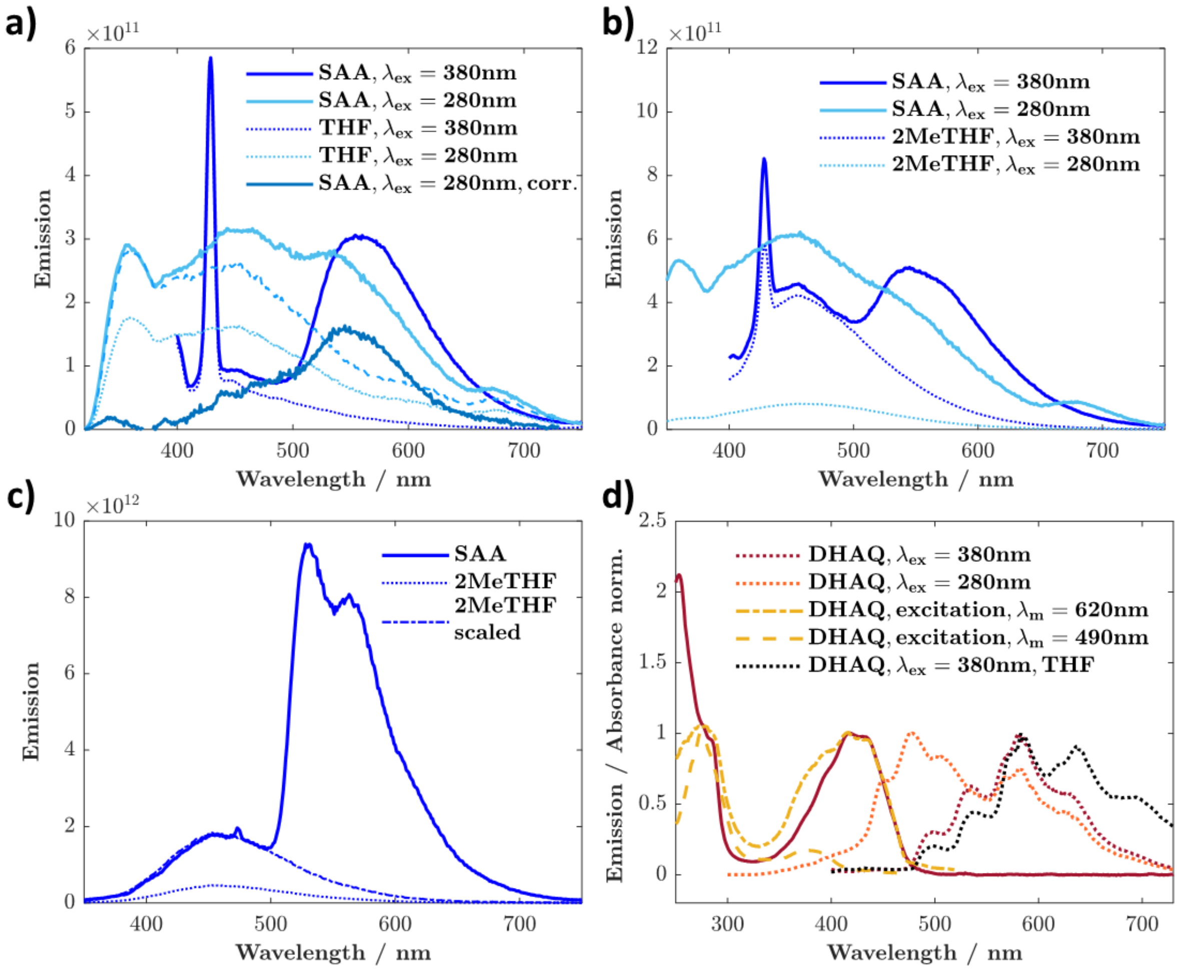

Figure S1. Emission data recorded at room temperature for SAA in a) THF, and b) 2MeTHF, together with the solvent backgrounds. In a) the corrected emission (solid blue, see legend) after subtraction of the scaled solvent background (dashed light blue) is also plotted. c) Emission data recorded at 77K for SAA in 2MeTHF, plotted together with the raw (dotted royal blue) and scaled (dashed royal blue) solvent backgrounds. Notice that, at 77K, the emission in the blue is suppressed / obscured entirely by the solvent background. d) Emission data recorded at 77K for DHAQ in 2MeTHF at the indicated excitation wavelengths. Excitation spectra monitored at the indicated wavelengths are also plotted. Note that the excitation spectrum monitored at 490 nm (dashed yellow) does not fully trace the lowest energy absorption band, potentially suggesting emission from a higher lying enol state (*vide infra*) is captured at this temperature. Emission data recorded at 77K for DHAQ in THF (dotted black) is also plotted for comparison, showing good agreement with the vibronic progression(s) observed in 2MeTHF. In general, the observed spectral features are very similar in both solvents. For room temperature measurements, excitation and emission slit widths (SWs) corresponding to a spectral resolution of 5 nm were used, and the integration time was 0.5 s. For 77K measurements, SWs of 2 nm and integration time of 0.2 s was used instead.

1.2. Quantum Yield Determination

Quantum yields were determined using relative actinometry, by comparing the integrated emission intensities of the samples and known standard[1], $[Ru(bpy)_3]^{2+}$, under identical measurement conditions. The following equation was then used to determine the quantum yield:

$$\phi_x = \phi_s \times \frac{I_x}{I_s} \times \frac{A_s}{A_x} \times \frac{\eta_x}{\eta_s}$$

ϕ_x is the quantum yield of the sample, ϕ_s is the known quantum yield of the standard, I_x and I_s are the integrated emission intensities of the sample and standard, while A_x and A_s are the absorbances at the excitation wavelength. The latter were kept close to 0.05±0.005 to permit for a linear correction of any differences. Furthermore, absorbances were kept <0.1 throughout the measurement window to prevent inner filter effects. Finally, the refractive indices η_x and η_s were approximated as those of the solvents for these dilute solutions. The quantum yield of SAA was determined using the same procedure, but with DHAQ as reference due to greater spectral similarity in the blue region and lower yields for the former.

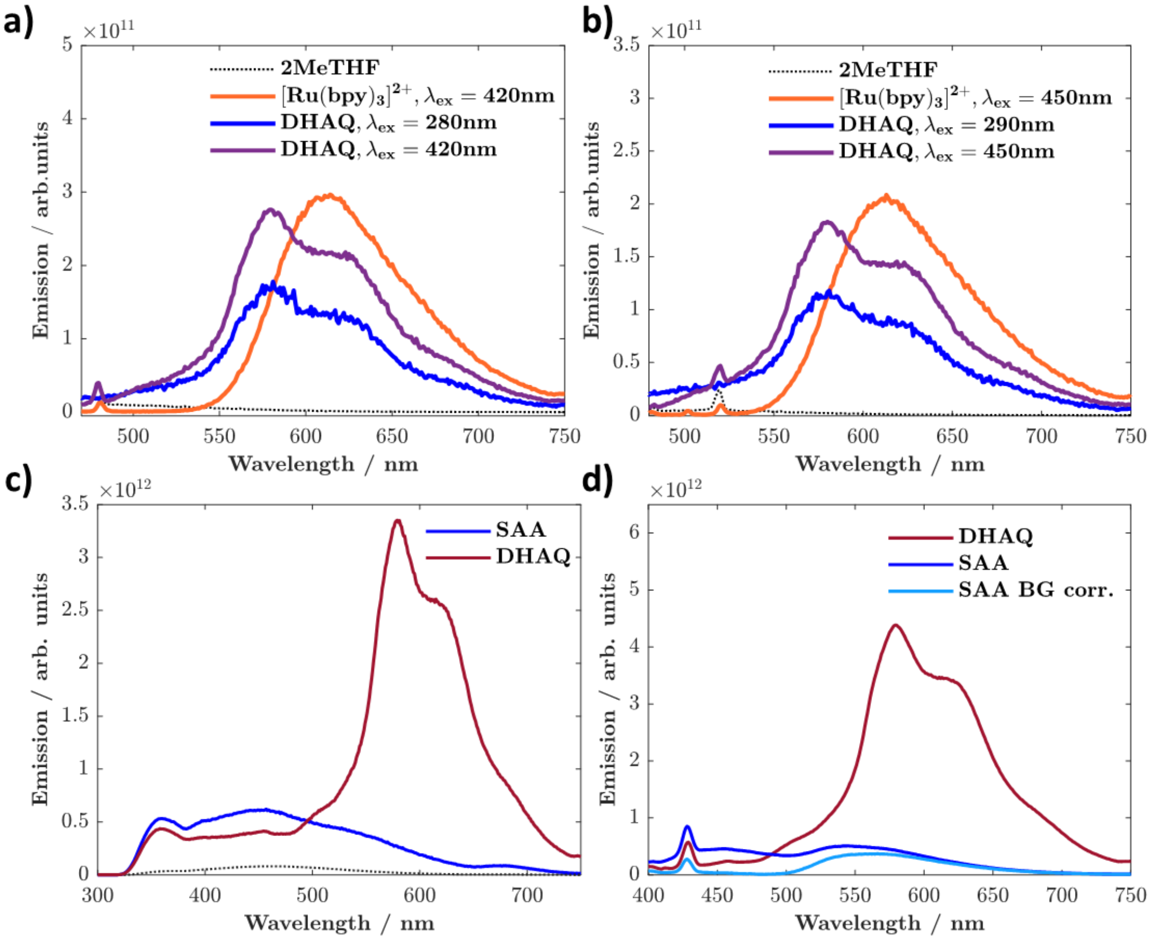

Figure S2. a) and b) Emission data recorded at room temperature for $[Ru(bpy)_3]^{2+}$ and DHAQ in 2MeTHF at the indicated excitation wavelengths, absorbance ≈ 0.05, excitation and emission SWs = 2 nm and integration time = 0.25 s. Emission data recorded at room temperature for SAA and DHAQ in THF, c) λ_{ex}=280 nm, and d) λ_{ex}=380 nm absorbance ≈ 0.05, excitation and emission SWs = 5 nm and integration time = 0.5 s.

2. Transient Absorption Spectroscopy

Global analysis of the data was carried out using the Glotaran software package[2], as previously described[3]. Briefly, a triexponential fit model was used, including convolution with the IRF, for the short timescale data. For longer timescales, data was truncated to eliminate effects from the coherent artifact at early times. Very similar results were obtained for truncation below 0.3 or 0.5 ps. Further fit parameter details are noted in the figures and associated captions below.

2.1. Global Analysis: SAA

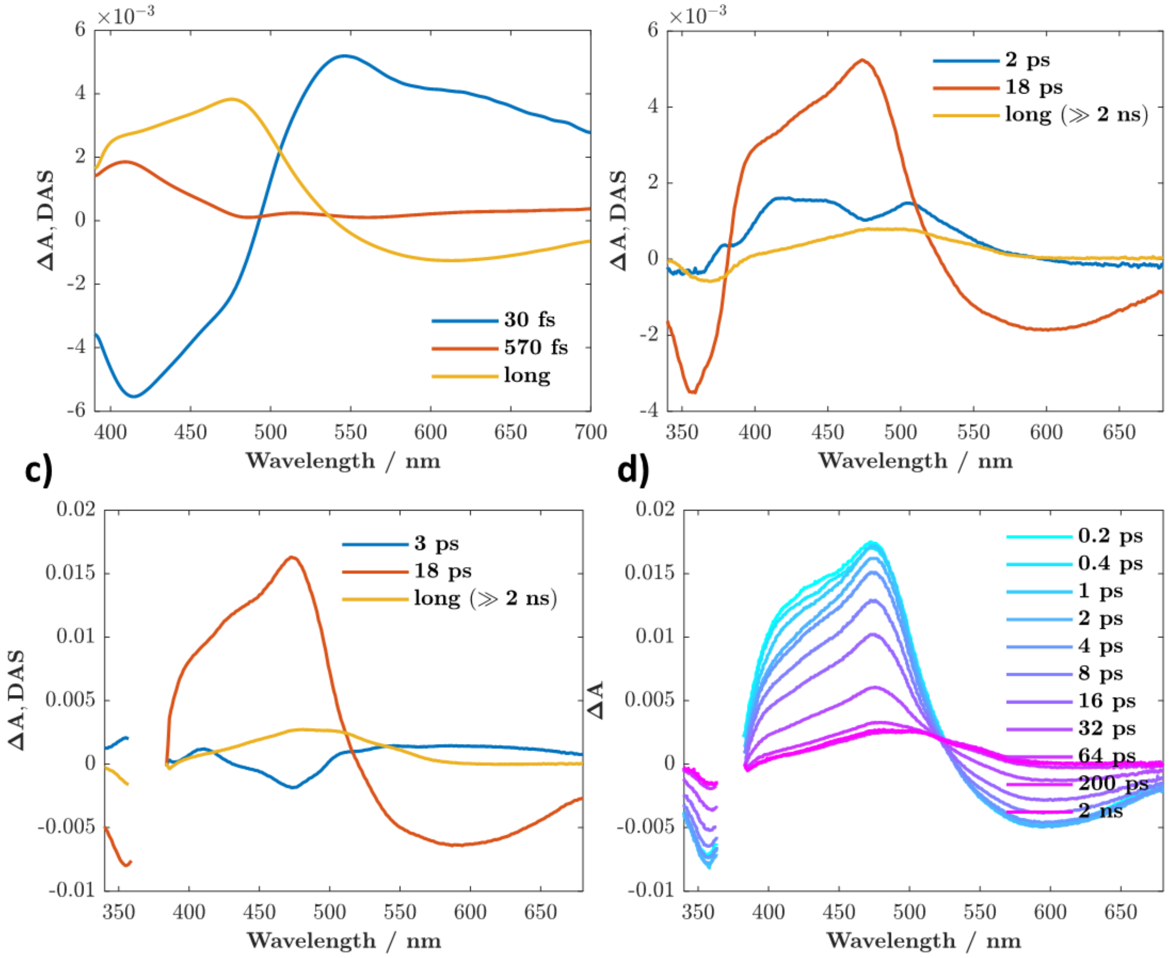

Figure S3. Decay associated spectra (DAS) extracted from global analysis of the TA data obtained after exciting SAA at 280 nm in THF. a) short, and b) long timescale data. All fit parameters were held free except the IRF width (~35 fs) and long component in a). c) DAS extracted from global analysis of the transient absorption data obtained after exciting at 380 nm on long timescales; data < 300 fs was truncated to avoid effects of the coherent artifact. Free fit parameters were 1.2 ps, 22 ps, and long. The keto recovery time was held fixed at 18 ps for a meaningful comparison with b) (shown). d) TA spectral data obtained after exciting SAA at 380 nm in THF.

2.2. Global Analysis: DHAQ

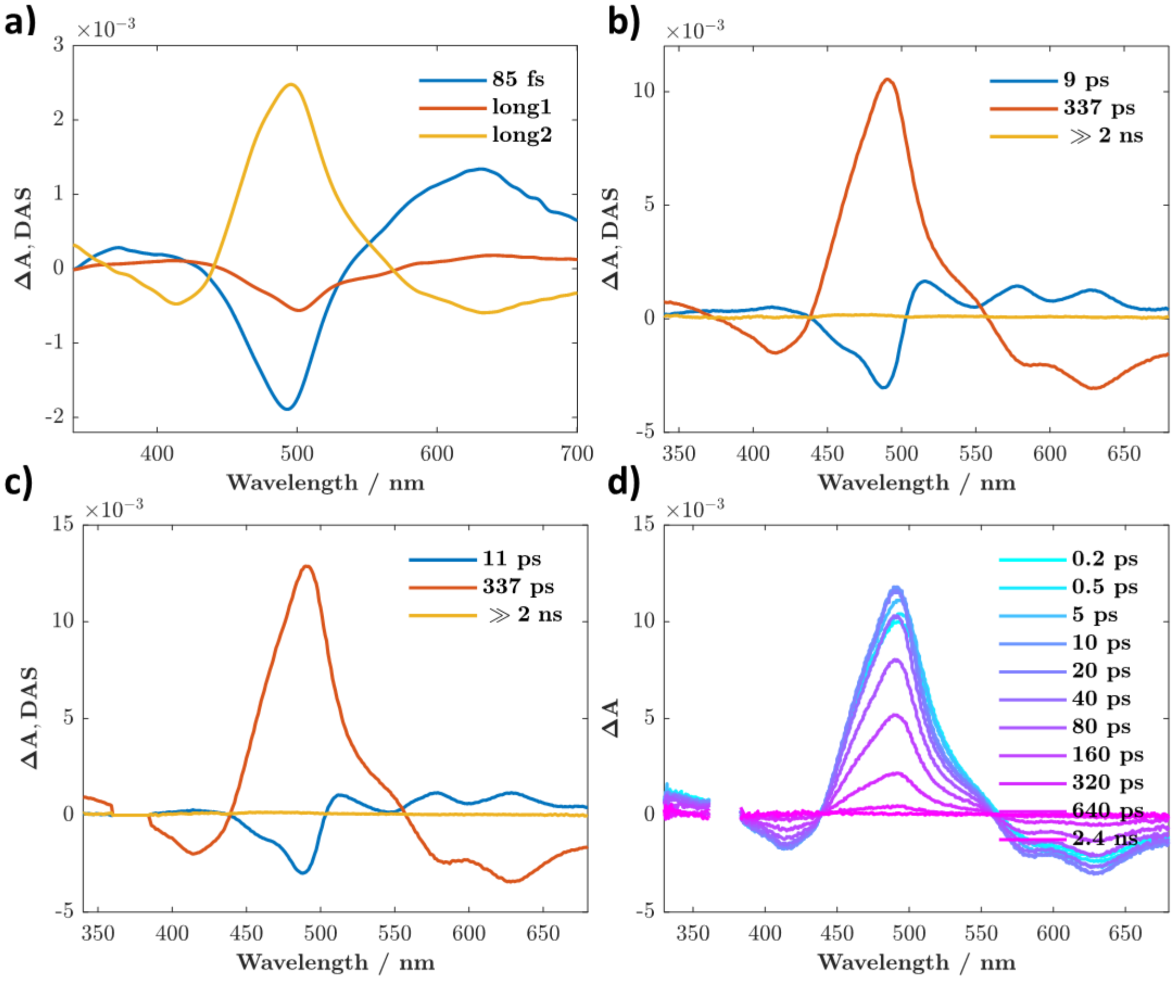

Figure S4. Decay associated spectra (DAS) extracted from global analysis of the TA data obtained after exciting DHAQ at 280 nm in THF. a) short, and b) long timescale data. All fit parameters were held free except the IRF width (~35 fs) and long2 component in a). Note that long1 and long2 are components with large time uncertainty, and only the ~85 fs component is well-resolved in the two picosecond delay window accessible in the experiment. The long(er) components are accurately resolved in the DAS associated with the long timescale data. c) DAS extracted from global analysis of the transient absorption data obtained after exciting at 380 nm on long timescales; data < 500 fs was truncated to avoid effects of the coherent artifact. Free fit parameters were 10.5 ps, 343 ps, and long. The keto recovery time was held fixed at 337 ps for a meaningful comparison with the shorter component in b) (shown). d) TA spectral data obtained after exciting DHAQ at 380 nm in THF.

3. Density Functional Theory (DFT) and Time-Dependent Density Functional Theory (TD-DFT)

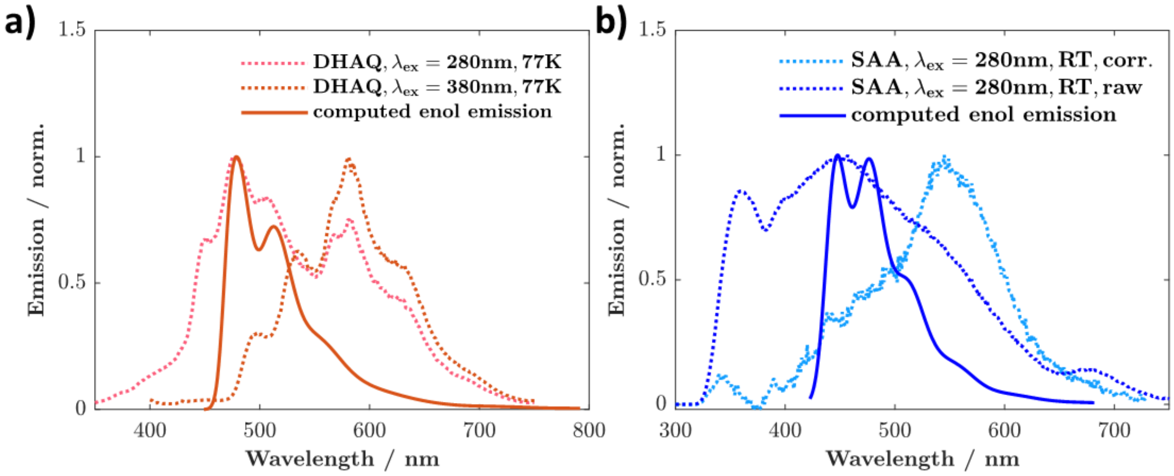

Figure S5. a) Comparison of the theoretically evaluated enol FCHT emission spectra and experimentally observed emission in DHAQ. b) Comparison of the theoretically evaluated enol FCHT emission spectra and experimentally observed emission in SAA. Note that the blue emission is obscured in the low temperature data, therefore room temperature emission is shown, precluding direct comparison of the vibrational progression(s).

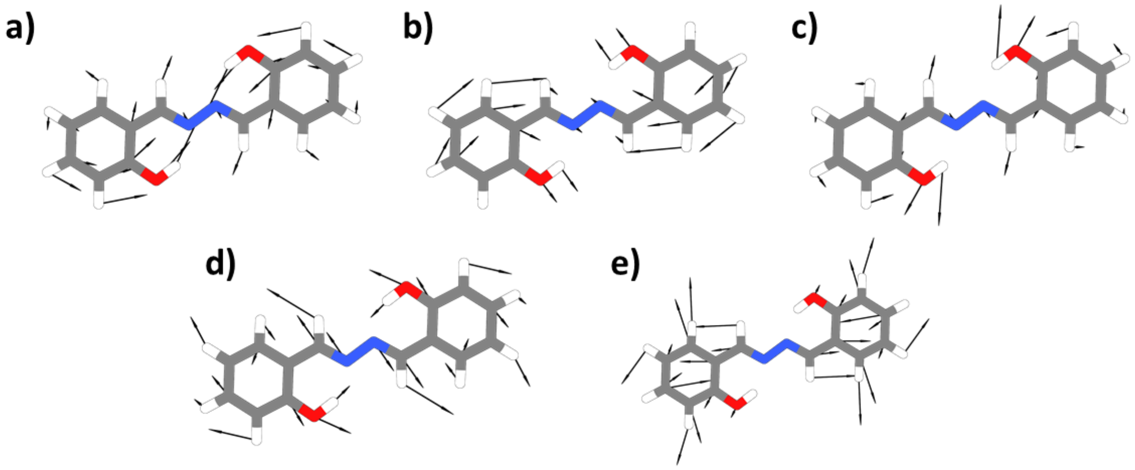

Figure S6. Computed vibrations for SAA: a) 142 cm^{-1}, b) 232 cm^{-1}, c) 445 cm^{-1}, d) 538 cm^{-1}, and e) 644 cm^{-1}.

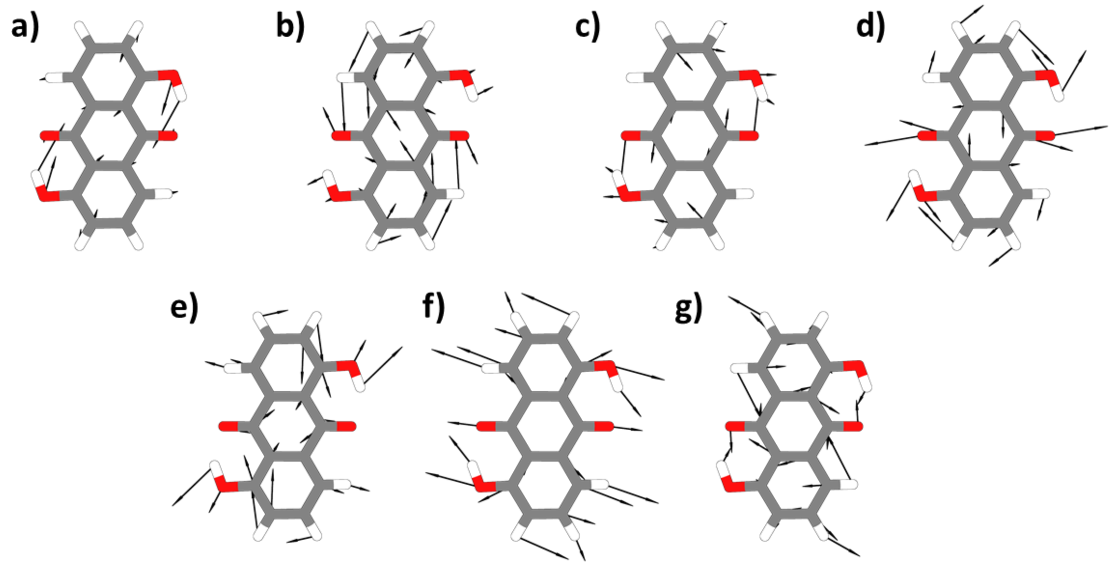

Figure S7. Computed vibrations for DHAQ: a) 302 cm^{-1}, b) 361 cm^{-1}, c) 400 cm^{-1}, d) 456 cm^{-1}, e) 513 cm^{-1}, f) 597 cm^{-1}, and g) 713 cm^{-1}.

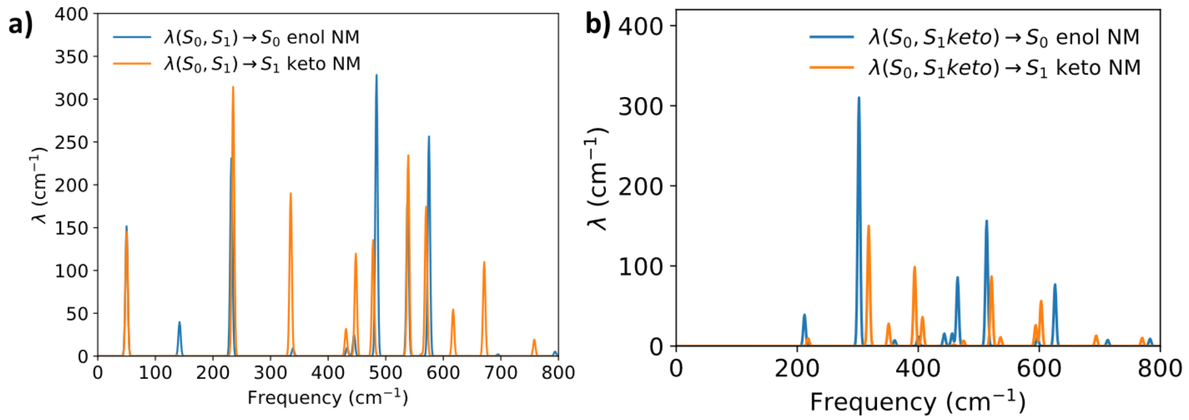

Figure S8. The calculated reorganization energies for ground state enol and S_1 state mono-keto geometries for a) SAA and b) DHAQ. The energies are calculated with both S_0 enol normal mode (NM) basis (blue solid line) and S_1 keto NM basis (orange solid line).

3.1. Tables

Table S1: Energy difference of optimized Monoketo and Diketo from Dienol optimized geometries for S_1 electronic states.

	DHAQ	SAA
$E_{Diketo} - E_{Dienol}$	0.107 eV (865.44 cm^{-1})	0.314 eV (2538.46 cm^{-1})
$E_{Monoketo} - E_{Dienol}$	-0.034 eV (-271.81 cm^{-1})	-0.134 eV (-1083.12 cm^{-1})

Table S2: The FCHT vibronic transitions for DHAQ absorption, with relative energy with respect to the 0-0 transition energy. The frequency (ω_n) corresponding to $|0\rangle \rightarrow |n\rangle$ transitions are reported.

Transition	Frequencies (cm^{-1})	Energy (cm^{-1})	Intensity
$\|0\rangle \rightarrow \|0\rangle$	--	0.00	0.12×10^5

$\|0\rangle \to \|9^1\rangle$	301.78	306.59	4393
$\|0\rangle \to \|11^1\rangle$	400.03	392.04	2834
$\|0\rangle \to \|13^1\rangle$	443.24	416.16	1402
$\|0\rangle \to \|19^1\rangle$	543.23	527.90	4613
$\|0\rangle \to \|19^1; 9^1\rangle$	543.23; 301.78	834.48	1759
$\|0\rangle \to \|53^1\rangle$	1392.51	1387.50	1379
$\|0\rangle \to \|61^1\rangle$	1616.32	1568.49	1390

Table S3: The FCHT vibronic transitions for SAA absorption, with relative energy with respect to the 0-0 transition energy. The frequency (ω_n) corresponding to $|0\rangle \to |n\rangle$ transitions are reported.

Transition	Frequencies (cm^{-1})	Energy (cm^{-1})	Intensity
$\|0\rangle \to \|0\rangle$	--	0.00	0.11×10^5
$\|0\rangle \to \|5^1\rangle$	142.06	148.82	0.13×10^5
$\|0\rangle \to \|8^1\rangle$	223.33	233.35	4173
$\|0\rangle \to \|5^2\rangle$	142.06	297.63	7892
$\|0\rangle \to \|8^1; 5^1\rangle$	223.33; 142.06	382.16	5094
$\|0\rangle \to \|5^3\rangle$	142.06	446.45	3367
$\|0\rangle \to \|8^1; 5^2\rangle$	223.33; 142.06	530.98	3218
$\|0\rangle \to \|60^1\rangle$	1410.59	1391.78	3263
$\|0\rangle \to \|62^1\rangle$	1445.77	1438.11	2283
$\|0\rangle \to \|63^1\rangle$	1485.91	1469.20	2472
$\|0\rangle \to \|60^1; 5^1\rangle$	1410.59; 142.06	1540.60	3952
$\|0\rangle \to \|62^1; 5^1\rangle$	1445.77; 142.06	1586.93	2843
$\|0\rangle \to \|63^1; 5^1\rangle$	1485.91; 142.06	1618.02	3085
$\|0\rangle \to \|60^1; 5^2\rangle$	1410.59; 142.06	1689.41	2478

4. References

(1) Ishida, H.; Tobita, S.; Hasegawa, Y.; Katoh, R.; Nozaki, K. Recent Advances in Instrumentation for Absolute Emission Quantum Yield Measurements. *Coordination Chemistry Reviews*. November 2010, pp 2449–2458. https://doi.org/10.1016/j.ccr.2010.04.006.

(2) Snellenburg, J. J.; Laptenok, S.; Seger, R.; Mullen, K. M.; van Stokkum, I. H. M. Glotaran: A Java-Based Graphical User Interface for the R Package TIMP. *Journal of Statistical Software; Vol 1, Issue 3 (2012)* 2012.

(3) Kaul, N.; Lomoth, R. The Carbene Cannibal: Photoinduced Symmetry-Breaking Charge Separation in an Fe(III) N-Heterocyclic Carbene. *J Am Chem Soc* 2021, *143* (29), 10816–10821. https://doi.org/10.1021/jacs.1c03770.
